# Emergency primary care personnel’s perception of professional-patient interaction in aggressive incidents -- a qualitative study

**DOI:** 10.1186/s12875-016-0454-7

**Published:** 2016-05-12

**Authors:** Tone Morken, Kjersti Alsaker, Ingrid H. Johansen

**Affiliations:** National Centre for Emergency Primary Health Care, Uni Research Health, Kalfarveien 31, 5018 Bergen, Norway; Faculty of Public Health and Social Sciences, Bergen University College, Box 7030, 5020 Bergen, Norway

**Keywords:** Qualitative research, Aggression, Workplace violence, Primary care, Professional-patient relations

## Abstract

**Background:**

Incidents of aggression and violence from patients and visitors occur in emergency primary care. Most previous studies have focused on risk factors such as characteristics of patient, health personnel or situation. This study aimed to explore professional-patient interaction in aggressive situations.

**Methods:**

A focus group study with eight focus groups was performed, including a total of 37 nurses and physicians aged 25–69 years. The participants were invited to talk about their experiences of violence in emergency primary care. Analysis was conducted by systematic text condensation. Results were then illuminated by Honneth’s theory The Struggle for Recognition.

**Results:**

We identified three main themes regarding the interaction between health personnel and patients or visitors in aggressive situations: (1) unmet needs, (2) involuntary assessment, and (3) unsolicited touch. In all interactions the aggressive behaviour could be understood as a struggle for recognition.

**Conclusions:**

Aggression is more likely to arise in situations where the patients’ needs or personal borders are invalidated. The struggle for personal recognition during the interaction between patient and health professionals should be addressed in health professionals’ education. This knowledge might increase their awareness and help them to react in a more expedient manner.

## Background

Working in emergency primary care is associated with high risk of experiencing aggression and violence from patients and visitors [1–3]. In Norway, municipalities are obliged by law to provide emergency primary care around the clock. This service is organised in special clinics or as part of a general medical practice, many of them small and isolated and far from the hospital, and not in hospital emergency departments as in many other countries. Depending on the size of the community served, the number of staff on duty at any given time varies from one to several persons, mainly physicians (mandatory) and nurses. The physicians primarily see patients at the clinic, but they also conduct home visits and participate on site in emergencies outside hospitals. When nurses or other health personnel are present, they perform triage in the patient’s initial contact with the centre, give advice when appropriate and assist the physician when needed.

Previous research on workplace violence in primary care has often focused on risk factors mostly related to characteristics of the patient, the general practitioner or the work environment [2–6]. In emergency primary care the patient is often unknown, and this limits the usefulness of warning signs related to the patient. However, aggression usually occurs as a result of interpersonal interactions [7, 8]. Cox and Leather [9] claim that “human aggression is typically the product of interpersonal interactions wherein two or more persons become involved in a sequence of escalating moves and counter moves, each of which successively modifies the probability of subsequent aggression”. Existing studies on the dynamics of the interaction between staff and patients have mostly been performed in psychiatric institutions and general hospitals [10]. The in-patient premises, settings and incidents differ in many respects from emergency out-patient settings [11], thus the transfer value of findings from in-patient settings is uncertain.

In a study exploring health personnel’s experiences with workplace violence [12], we were struck by how most narratives contained rich and detailed descriptions of the interaction that took place before and under the incident. Although a multitude of factors make each professional-patient interaction unique, an analysis of the experienced interaction may give valuable information about generic situations in which violence could be anticipated. The aim of this study was therefore to explore the emergency primary care personnel’s perception of professional-patient interaction in aggressive situations. An aggressive situation is in this study defined as a situation where the professional experiences verbal abuse, threats or physical abuse from the patient or visitor.

## Methods

This study was based on a re-reading of a data material, collected in a focus group study, which explored emergency primary care staffs’ experiences with workplace violence [12].

### Participants

The focus group study was performed among health care personnel with work experience from Norwegian emergency primary care. Participants were recruited by announcement at conferences and in an educational program for registered nurses specializing in emergency primary care, and by e-mails distributed to employees via managers at emergency primary care centres in different parts of Norway. Some participants were recruited through other participants. Initially the main criterion for inclusion was personal experience of threats or violence, and the potential participants were invited to contact the researchers directly by phone or by email. However, this strategy mostly recruited nurses. To access experiences of general practitioners (GPs), an open invitation was sent to pre-established collegial discussion groups. The GP groups were not given any inclusion criteria apart from willingness to discuss the issue. A relatively large sample of physicians were recruited to increase the chance of talking to physicians who had experienced aggressive situations, and to ensure coverage of different lengths of work experience and experience from different organisational models and populations [13]. A total of 37 physicians and nurses were included in the study (Table 1). There was a slight majority of physicians and females. Mean age was 41 years (range 25–69). Mean length of work experience in emergency primary care was 9 years (range 1–33). The participants had work experience from an organisationally and geographically diverse subset of emergency primary care clinics.

**Table 1:** Sample distribution (*n* = 37)

		Number	Percent
Occupation	Nurse	15	41
	Physician	22	59
Gender	Female	23	62
	Male	14	38
Age	18–29 years	3	8
	30–39 years	18	49
	40–49 years	8	22
	50–59 years	6	16
	>59 years	2	5
Years worked in emergency primary care			
	1–5 years	18	49
	6–10 years	8	21
	>10 years	11	30

### Data gathering

Eight focus groups were convened in the period between October 2012 and November 2013. Each group comprised two to six participants with similar professions, and everybody participated in one focus group discussion only. Three of the groups were pre-established GP groups. Nobody dropped out after inclusion in the study. The focus group were performed at participants’ workplace or at one of the participants’ home. Nobody was present besides the researchers and participants. Before the focus group discussion started, all participants gave a written informed consent to the secretary (KA or TM) of the focus group. The participants were also asked to complete a brief form, including questions on age, occupational title and years of work experience in emergency primary care. All discussions were recorded by digital sound recorder, and field notes were taken under and after the meeting. On average, the discussions lasted approximately 90 min, and each group had one meeting. The moderator (IHJ or TM) initiated the discussions by presenting themselves and the reasons for doing the research and they then invited everyone to talk about personal experiences of threats or violence. A short interview guide was made in advance and the discussion was structured around the question “Can you describe a personal experience of threats or violence”. The group members were encouraged to talk freely and comment on one another’s stories. Focus groups were conducted until the researchers no longer received new information and the sample seemed sufficiently large to elucidate the initial aim of the study.

### Analysis

Each interview was transcribed verbatim by TM or IHJ. The transcripts were audited by a co-researcher (IHJ or TM) for reliability, and later imported into a qualitative software package (Nvivo 10) to aid data analysis. The analysis process was based on systematic text condensation, which is a descriptive approach focusing on the experiences expressed by the participants themselves, rather than exploring possible underlying meanings. Systematic text condensation is rooted in phenomenology, and stresses the importance of ensuring that the analysis is guided by the actual data, instead of predefined theoretical frameworks [14]. In the present study, the transcripts were read by all authors focusing on the description of interactions between health personnel and patients or other visitors in aggressive situations. Emerging themes were discussed. Based on these themes, meaning units were identified and coded, representing different aspects of the participants’ described interactions. The contents of each coded group were condensed, and then summarized to make generalised descriptions of the interactions. The final description was illustrated by selected quotations. All quoted participants were given pseudonyms. Transcripts were not returned to participants for comments or correction. In parallel with refining the summaries of the coded groups, relevant philosophical theories were explored. The theory Struggle for Recognition by Axel Honneth [15] seemed to give a deeper understanding of the findings, and was therefore included as a reference point in the discussion. All authors are female, health workers and PhD and all have performed research studies including focus group studies previously. One of the authors (IHJ) is a GP with clinical experience from emergency primary care.

## Results

In the descriptions of interactions between health professionals and patient or visitors in aggressive situations, we identified three main themes: (1) unmet needs, (2) involuntary assessment, and (3) unsolicited touch.

### Unmet needs

Several participants told stories about patients who attended the emergency primary care centre with needs that the health personnel did not comply with in the way they perceived that the patient, or relative, expected. The conflict sometimes escalated to a situation that the participant described as aggressive. Typically, the physician or nurse defined the presented problem as a minor ailment or a condition which did not require immediate medical care, and was not therefore something they were supposed to deal with. Thus they recommended the patient to contact their GP instead. This kind of message seemed to increase the patient’s frustration, and the situation might culminate in verbal abuse or even physical violence against the nurse or the physician. Examples of participant’s stories were: A patient attending the clinic with fever and symptoms that the professional considered was a common cold, advising her to contact the regular general practitioner (rGP) the next day; a patient with low abdominal pain arriving at the clinic by ambulance, then having to wait for an hour before being attended to by a physician; or a patient contacting the clinic to get painkillers, and being denied this by the physician.

In a number of stories participants reflected on how health personnel themselves may contribute to the development of an encounter becoming aggressive by their behaviour. A nurse described how she had observed colleagues delivering unpleasant messages in a pleasant manner, and how this had made her try to modulate her own high-pitched voice to talk more soothingly. In another story a physician reflected on how certain situations often resulted in conflict. He gave an example in where a well-educated couple attended the emergency primary care center with their feverish child. After examining the child, the physician concluded that no medical treatment was needed. The parents then presented their child’s chronic rash and said that they wanted the physician’s opinion, but were denied this. The situation escalated:

*…they insisted on my professional judgement regarding the diagnosis and treatment (of the rash), and they claimed that this was their right according to the Norwegian Health and Rights Act, and that they were lawyers. It was a hectic day, with lots of children to attend to and some very ill children. And I react this way when resourceful people demand something which I consider unreasonable. I become very spiky, a discussion evolves and – yes. It just turns unpleasant. (Tom, physician)*

### Involuntary assessment

Participants talked about incidents of threat or violence that occurred in the context of an enforced clinical assessment. Examples of such incidents were relatives asking for help and told they were worried about the patient’s mental health, the police demanding a blood test to determine if a patient had been intoxicated whilst driving, or the police bringing the patient to the clinic for a medical or psychiatric evaluation. Although some of these stories culminated in physical violence towards the health personnel involved, most of the stories described how the health personnel managed to deescalate and calm down a situation which initially had been threatening.

In several of the stories the participants told that they actively tried to build an alliance with the patient, or avoided overt conflict by being more flexible in their approach, or by presenting solutions which restored or increased the patient’s dignity. A nurse told about how she experienced that a patient calmed down when she recognized his psychological pain instead of confronting his aggressive behavior. In another story a physician was called out to the police station to take a blood sample of an agitated prisoner who refused to comply with the police’s orders:

*And then I asked if I could talk to him alone. The police left the cell, and I told him that they (the police) would not give in and that the situation would turn into a terrible racket. I asked him: Should we just do it safe and simple without trouble? Okay, he said. And then we went out together, into the locker room which had better lighting. And he presented his arm, and the blood specimen was collected, and the police remained far away in the background. (Liam, physician)*

### Unsolicited touch

Some of the participants told stories where the violent act obviously had been preceded by the health personnel touching the patient. Typically the patient had been sitting in the consulting or waiting room, and the physician or nurse had observed her as being anxious or tense. The professional had then approached the patient, talking to and touching the patient to calm her down or to express sympathy or care. The touch sometimes preceded eye-contact or other more formal contact with the patient. A nurse described the interaction with a young girl, who was sitting crouched in the consulting room, together with two ambulance personnel, waiting for the physician:

*… I entered the room from a door behind (the patient). Then I put my arm around her like this (demonstrates) and said «you will soon receive help». That was a stupid thing to do. It triggered something in her. The little girl - she jumped up and tried to strangulate me while she pushed me up and towards the wall. (Ann, nurse)*

Through these stories participants reflected on how they in retrospect understood how their well-meant, but enforced, intimacy had infringed upon the patient or possibly triggered a previous trauma. They also problematized the general teaching of showing care by touching, and suggested that the patient needed to be prepared for physical contact.

## Discussion

This study suggests that professional-patient interactions which include unmet needs, involuntary assessment and unsolicited touch may trigger aggression. Encounters between health personnel and patients are intrinsically asymmetrical, with uneven distribution of power. The health personnel are the key to something the patient needs, and they therefore hold power in the interaction. Thus, the main responsibility for a decent interaction lies with the health professional. Recognizing interactions with increased risk of aggression might help the health personnel prevent aggressive incidents.

### Aggression as struggle for recognition

In the theory Struggle for Recognition, Honneth describes how non- and misrecognition can become a potential motivator for interpersonal conflicts [15]. He claims that within a social interaction, each individual needs to be recognized by the other(s) to preserve their self-esteem. Aggression can thus be perceived as a demand for rights (legal relations) and a demand for recognition as a unique person. This understanding adds to the comprehension of our main findings, and we will therefore discuss the findings in light of Honneth’s theory and existing literature.

Honneth describes several types of disdain which might affect the individual’s self-esteem. Firstly, an exclusion from what the individual considers to be his or her right, can be perceived as a humiliation, and therefore inflict damage to the person’s self-respect [15]. When an experienced need is not met, this could be perceived as social contempt, disrespect or unjust, which would then be accompanied by anger [16]. Several studies have identified unmet needs as a trigger of conflicts [17–19]. However, the perception of injustice does not necessarily arise from a refusal in itself, but when the patient’s perspectives and understanding of the problem are not emphasised at all [16]. In line with this, studies have reported that positive encounters with aggressive clients were characterized by a mutual recognition between caregiver and client [20], and that patients might react more to the way rules are communicated and enforced, than to the actual rules [21]. Furthermore, feeling ignored, misunderstood or misinterpreted seems to be particularly provoking [21]. In emergency primary care aggressive incidents might therefore be reduced by paying more attention to the expectations and needs of patients [22], and by improving the health personnel’s ability to acknowledge these needs in situations where the needs cannot be met.

Secondly, Honneth claims that being deprived the right to decide over your own body is the strongest and most fundamental form of personal debasement [15]. He also claims that “what is called ‘human dignity’ may simply be the recognizable capacity to assert claims” [15]. In involuntarily assessments, the patients are deprived the right to decide over their own bodies. Furthermore, the patient may be expected to supress the otherwise normal response of trying to defend oneself if physically attacked or restrained [23]. This makes the patient subordinate and extremely vulnerable. A study in a hospital found that enforced personal care and medical treatment on apparently reluctant recipients were related to aggressive incidents [10]. It might therefore be that the aggression can be modulated by health professional overtly recognising injustice to the patient and trying to help them restore their dignity in the situation.

In a similar manner to involuntary assessment, unsolicited touch can be understood as an abuse or violation against physical integrity. A previous study found that physical contact initiated by health personnel was a stimulus which sometimes preceded assaults [24]. Health personnel should therefore be made aware that although touching is an established way of conveying care, it could also be experienced as an invasion or a strong reminder of previous trauma. They should therefore be attentive when they approach a patient physically.

### Study strength and limitations

We received several stories from different parts of Norway, describing various situations of threats or violence at work in emergency primary care. We consider the material to represent a broad range of experiences among emergency primary care personnel. A limitation to our study is that all authors are females, which might have influenced the observations and interpretations. Another limitation is that the focus group study included only health personnel, and that all narratives are told from their perspective. It has been shown that staff and patients have different perceptions of aggressive incidents and causes of aggression, and that some interpersonal factors important to patients were not mentioned by staff [25, 26]. Hence, we may lack relevant aspects of the patient-professional interaction [23]. In addition, health professionals’ interaction with patients and their communication skills are potential sensitive and emotive topics. The stories and versions told by health professionals in our study may therefore be influenced by the need for showing themselves in a favourable light. Furthermore, our study was originally designed to explore health personnel’s experiences, focusing on organisational factors [12]. Although the richness of the existing data on interpersonal interactions was one of our main reasons for pursuing this topic, the study’s original focus might have affected the findings.

## Conclusions

Aggression in interactions between health professionals and patients was described in situations with unmet needs, involuntary assessment and unsolicited touch of the patient. The aggression in these situations can be understood as a result of the patient’s struggle for recognition.

The struggle for recognition in the interaction between patient and health professionals should be addressed in health professionals’ education. Educating health professionals about types of interaction in which aggression is more likely to arise, might increase their alertness and help them to react in a more expedient manner. Using stories of aggressive incidents as a tool for reflective practice could be one way of preparing health professionals for aggressive encounters.

## Ethics approval

The study was approved by the Regional Committee for Medical and Health Research Ethics.

## Informed consent

We confirm that all patient/personal identifiers have been removed or disguised so the patient/persons described are not identifiable and cannot be identified through the details of the story.
